# Continuous Spinal Anesthesia for Emergent Laparotomy in a Patient With Severe Pulmonary Comorbidities: A Case Report

**DOI:** 10.7759/cureus.74683

**Published:** 2024-11-28

**Authors:** Ana Patrícia Pereira, Francisco Teixeira, Elisabete Pereira, José Carlos Sampaio, Catarina Sampaio

**Affiliations:** 1 Anesthesiology Department, Unidade Local de Saúde de Trás-os-Montes e Alto Douro, Vila Real, PRT; 2 Anesthesiology Department, Unidade Local de Saúde de Gaia e Espinho, Vila Nova de Gaia, PRT

**Keywords:** chronic obstructive pulmonary disease, continuous spinal anesthesia, laparotomy, prilocaine, spinal anesthesia

## Abstract

Severe pulmonary conditions in patients undergoing surgery place them at greater risk of adverse outcomes. Alternatives to general anesthesia are encouraged, particularly for emergent interventions, as these procedures can compromise the patients' full recovery postoperatively. We describe an example of an underused anesthetic technique, continuous spinal anesthesia, to circumvent the perioperative risk of major abdominal surgery, successfully avoiding invasive mechanical ventilation in these patients.

## Introduction

A growing number of patients are presenting for surgery with multiple comorbidities, which place them at a superior risk of an adverse outcome [1]. General anesthesia (GA) with endotracheal intubation is routinely employed for major abdominal surgery. Although GA provides controlled ventilation and muscle relaxation, it is linked to pulmonary atelectasis and other respiratory complications, which may contribute to acute respiratory failure and the need for postoperative mechanical ventilation [2]. This risk is known to be increased in individuals with pulmonary comorbidities and may lead to prolonged hospitalization, morbidity, and mortality [3,4]. An alternative anesthesia strategy may avoid further respiratory compromise in these already frail patients [5].

Continuous spinal anesthesia (CSA) is a well-established although underutilized technique in modern anesthesia practice. It may have advantages over other neuraxial anesthetic choices owing to its ability to combine the reliable characteristics of a subarachnoid block with the possibility of titration and the ability to extend the anesthetic duration of a catheter technique [1,3,6-8]. The use of regional anesthesia in the operative setting may preserve patient respiratory function and provide effective blockade of the stress response, and CSA allows greater control over surgical anesthesia than an epidural block, with predictable effect and less hemodynamic instability than a single injection subarachnoid block [3,9].

Despite some controversy about its use, CSA has been applied in complex patients undergoing vascular, orthopedic, and general surgery [9,10]. A case series described the successful use of CSA as the primary method of anesthesia for elective colorectal cancer surgery in 68 high-risk patients over 14 years and suggests it can avoid ICU admission in these patients [3]. We present a case in which CSA is used as a primary choice of anesthesia in a patient with severe pulmonary comorbidity undergoing major abdominal emergency surgery.

## Case presentation

A 73-year-old man visited the emergency department with severe diffuse abdominal pain and a 15-day history of constipation. Medical history included poorly controlled arterial hypertension, mild aortic insufficiency, severe chronic obstructive pulmonary disease (COPD) (grade III, group D), pulmonary emphysema, bronchiectasis, squamous cell lung carcinoma clinical staging T1cN2M0 IIIA (never gathering surgery, radio or chemotherapy conditions) under continuous domiciliary oxygen therapy (2L/minute), and a recent (<2 weeks) hospitalization for bacterial pneumonia with respiratory failure. On examination, he was agitated and hypotensive, with a mottling score of 2. Laboratory investigation results are given in Table 1.

**Table 1:** Relevant laboratory findings and reference values CKD-EPI: Chronic Kidney Disease Epidemiology Collaboration

Parameters	Patient Values	Reference Range
Hemoglobin (g/dL)	16.0	13.0-18.0
Leukocytes (x 10^3^/uL)	13	4.0-11.0
Platelets (x 10^3^/uL)	110	150-400
Prothrombin time (secpnd)	14.1	
International normalized ratio	1.03	<1.2
Activated partial thromboplastin time (second)	28.9	24-35
Reactive C protein (mg/dL)	31.7	<0.5
Serum creatinine (mg/dL)	1.7	0.7-1.4
Glomerular filtration rate (CKD-EPI) (mL/minute/1.73 m²)	38.98	
Sodium (mEq/L)	123	135-147
Potassium (mEq/L)	5.3	3.7-5.1
Total bilirubin (mg/dL)	1.7	<1.2
Lactates (mmol/L)	1.2	0.5-1.6

**Figure 1:**
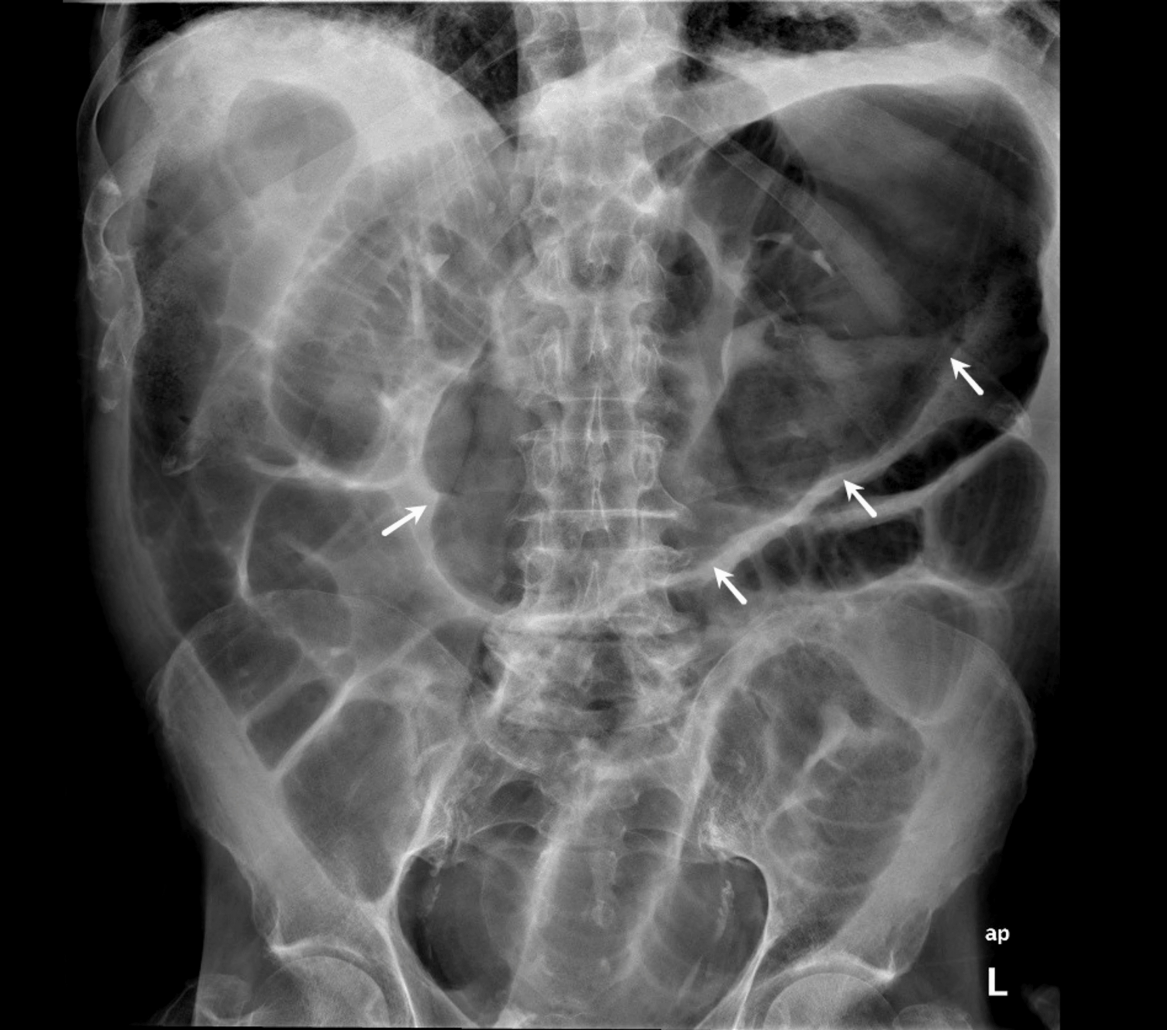
Abdominal X-Ray (erect anteroposterior view) suggesting a right colonic/cecal volvulus Visible: large gas-filled viscus on the left side of the abdomen, distal colon apparently empty, marked gaseous distension of small bowel loops

**Figure 2:**
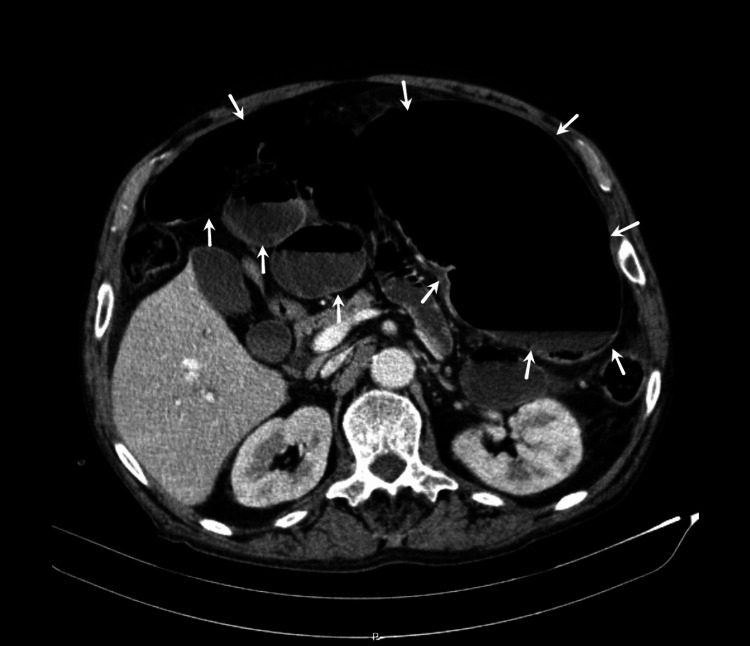
Abdominal CT scan (axial plane view) with major colon distension and air-fluid levels

Imaging with abdominal radiography and CT showed an apparent right colonic/cecal volvulus diagnosis (Figure 1, 2).

A decompression colonoscopy was attempted but failed to resolve it. An emergent exploratory laparotomy was proposed to solve the volvulus, which was starting to originate shock signs, and the patient presented to our operating room.

The patient was classified as American Society of Anesthesiologists (ASA) IV and his ARISCAT (Assess Respiratory Risk in Surgical Patients in Catalonia) score was 67 points (high risk), with 42.1% risk of pulmonary postoperative complications. The co-existing disease, its severity, and the physiological reserve of the patient also limited his admission to the ICU for the postoperative period. A CSA was chosen as the anesthetic technique and the patient’s consent was obtained after careful explanation. Perifix® epidural set (B. Braun SE, Melsungen, Germany) was used for the technique. Dural puncture was made at L2-L3 level, using an 18G Tuohy needle, and the catheter was introduced 3cm intrathecally. Small doses of prilocaine 2% slowly titrated to a total of 50 mg were injected through the catheter (20 mg at first and then 10 mg bolus 5 minutes apart) and a satisfactory sensory blockade was achieved at T4-T5, tested with mechanical stimuli (touch and skin pinch), cold and pinprick. The coincident hemodynamic repercussions were easily treated with fluids and a small bolus of phenylephrine as needed, to maintain mean arterial pressure above 65-70 mmHg. A total dose of 400 mcg was used.

Intraoperative monitoring included O2 peripheral saturation, 5-lead electrocardiogram, invasive blood pressure, and urine output. An infusion of 0.7 mcg/kg/hour of dexmedetomidine was initiated right after the neuraxial technique and maintained for patient comfort. The surgical procedure consisted of a xipho-pubic incision, right hemicolectomy, and laterolateral ileocolic anastomosis and lasted for 50 minutes. Surgeons found mild content of free serous fluid in the abdomen, a cecal and ascending colon volvulus with exuberant >15 cm diameter distension, and signs of bowel loop ischemia. Throughout the procedure, there was a further requirement of 20 mg of prilocaine. The patient remained hemodynamically stable and O2 peripheric saturation maintained above 94% while receiving O2 at 3L/minute by nasal cannula. In the end, 100 mcg of morphine was administered intrathecally, and the spinal catheter was removed. The patient was comfortable after surgery and safely discharged from anesthesia care to the ward after a two-hour stay in the post-anesthesia care unit (PACU). The motor block recovery was noted 40 minutes after the last prilocaine dose, and evidenced by the movement of feet and toes.

An endovenous regimen was elected for postoperative analgesia and consisted of paracetamol 1 g 6/6h and a 150 ml reservoir drug infusion balloon (DIB) at 3.1 ml/hour, with tramadol 600 mg, metamizole 8000 mg, and metoclopramide 60 mg, initiated two hours after the procedure, at the end of the stay in the PACU. Rescue analgesia included endovenous morphine 2 mg maximum 4/4h. The patient was referred to the acute pain unit which visited him for the next two days. No rescue analgesia was needed and no evidence of complications attributable to the anesthetic technique occurred at that point. However, the patient's postoperative course was further complicated by mild nosocomial pneumonia and pleural effusion treated with broad-spectrum antibiotics, non-invasive ventilation (with continuous positive airway pressure), and bronchodilators. The patient was discharged home on the 12th postoperative day.

## Discussion

This case demonstrates the successful use of CSA as a viable alternative to GA in a high-risk patient undergoing an emergent major abdominal surgery. The patient, with severe COPD, a history of lung cancer, and recent pneumonia, was at significant risk for postoperative pulmonary complications if submitted to GA. The consideration of co-existing severe respiratory disease and low physiological reserve limiting the possibility of patient’s admission to ICU, and the need to avoid postoperative mechanical ventilation, coupled with the coincident constrain with advanced postoperative care beds in the ICU, further dictated the need for an alternative anesthetic approach, considering the urgency of the procedure [5,9]. CSA combines the advantages of single-dose spinal anesthesia, which has a rapid onset and a high degree of success, with those of a continuous technique [6,7]. Besides providing the ability to extend the duration of anesthesia, it offers the ability to titrate doses of local anesthetic which allows precise control of the sensory block, minimizing the risk of hemodynamic instability [1,8,10].

Here, the use of prilocaine, a less commonly chosen local anesthetic when performing CSA, proved to be an effective decision. Its favorable safety profile and short duration of action facilitated a stable intraoperative course [6]. CSA's use in patients with severe pulmonary conditions is supported by the existing literature, which describes its benefits in reducing respiratory complications. Wong et al. [4] highlighted that patients with COPD may be more prone to postoperative pulmonary complications and Hausman et al. [11] stated that regional anesthesia in patients with COPD is associated with a lower incidence of postoperative respiratory failure. Similarly, an analysis by Hutton et al. found that regional anesthesia techniques can decrease the risk of postoperative pulmonary complications compared to GA [12].

The addition of dexmedetomidine provided sedation and analgesia while maintaining respiratory function and stable oxygen saturation levels throughout the procedure, also contributing to patient comfort [13].

This case underlines the importance of individualized anesthetic planning, particularly in patients with multiple comorbidities and limited postoperative care resources. The successful outcome in this patient highlights CSA as a valuable technique in managing high-risk surgical patients, providing effective anesthesia while mitigating the risk of respiratory complications, and avoiding the need for ICU admission [3]. Despite its advantages, CSA remains underutilized, mainly due to concerns about the required technical expertise and potential complications like the cauda equina syndrome, which was reported in the past with the use of microcatheters and high concentrations of lidocaine [1,3,14]. The safety of CSA is nowadays reinforced by recent advancements in the catheter/needle design and the local anesthetic's safety. However, the use of an epidural catheter is a safe option as demonstrated by this case and reported in the literature [7,8,15].

Increasing familiarity and training in CSA techniques among anesthesiologists could enhance and expand its use, particularly in high-risk populations where GA poses significant risks. Education and simulation training have been shown to improve proficiency in neuraxial techniques, which could help mitigate concerns and increase the adoption of CSA [16-19].

## Conclusions

CSA anesthesia remains a useful anesthetic alternative for abdominal surgery in patients with significant pulmonary comorbidities, who are at substantial risk for postoperative complications. This technique allows precise control of the level, intensity, and duration of spinal anesthesia, minimizes the risk of sudden hemodynamic collapse, and avoids further respiratory compromise in frail patients, making it a valuable tool in the anesthetic arsenal, particularly in settings with limited postoperative care resources. It may prevent the need for intensive postoperative monitoring and mechanical invasive ventilation, optimizing care without compromising patient safety. Further studies and case reports are encouraged to validate and expand upon these findings, promoting wider adoption of CSA in appropriate clinical scenarios.
